# Validating the ADFSCI hypotension symptom domain as a scalable patient reported outcome measure in spinal cord injury

**DOI:** 10.1038/s41746-026-02841-w

**Published:** 2026-06-01

**Authors:** Wenbin Yang, Meagan N. Smith, Julien Rimok, Aasta P. Gandhi, Nicolas Hankov, Nicolò Macellari, Anouk E. J. Nijland, Ilse J. W. van Nes, Léonie Asboth, Jordan Squair, Jocelyne Bloch, Nelleke G. Langerak, Noël L. W. Keijsers, Erkan Kurt, Sungchul Huh, Kelly A. Larkin-Kaiser, Jill M. Wecht, Stephen L. McKenna, Grégoire Courtine, Aaron A. Phillips

**Affiliations:** 1 Department of Physiology and Pharmacology, Cumming School of Medicine, University of Calgary, Calgary, AB Canada; 2 Department of Clinical Neurosciences, Hotchkiss Brain Institute, Cumming School of Medicine, University of Calgary, Calgary, AB Canada; 3 Department of Cardiac Sciences, Libin Cardiovascular Institute, Cumming School of Medicine, University of Calgary, Calgary, AB Canada; 4 Restore Network, Hotchkiss Brain Institute, Libin Cardiovascular Institute, McCaig Institute for Bone and Joint Health, Cumming School of Medicine, University of Calgary, Calgary, AB Canada; 5 Department of Biomedical Engineering, Schulich School of Engineering, University of Calgary, Calgary, AB Canada; 6 Defitech Center for Interventional Neurotherapies (NeuroRestore), CHUV/UNIL/EPFL, Lausanne, Switzerland; 7 Department of Clinical Neuroscience, Lausanne University Hospital (CHUV) and University of Lausanne (UNIL), Lausanne, Switzerland; 8 NeuroX Institute, School of Life Sciences, Swiss Federal Institute of Technology (EPFL), Lausanne, Switzerland; 9 Department of Research, Sint Maartenskliniek, Nijmegen, The Netherlands; 10 Department of Medical BioSciences, Radboud University Medical Center, Nijmegen, The Netherlands; 11 Department of Spinal Cord Injury Rehabilitation, Sint Maartenskliniek, Nijmegen, The Netherlands; 12 Department of Neurosurgery, Lausanne University Hospital (CHUV) and University of Lausanne (UNIL), Lausanne, Switzerland; 13 Department of Neurosurgery, Radboud University Medical Center, Nijmegen, The Netherlands; 14 Department of Rehabilitation Medicine, Pusan National University Yangsan Hospital, Yangsan, Republic of Korea; 15 Research Institute for Convergence of Biomedical Science and Technology, Pusan National University Yangsan Hospital, Yangsan, Republic of Korea; 16 Spinal Cord Injury Research Service, James J. Peters VA Medical Center, Bronx, NY USA; 17 Department of Rehabilitation Medicine and Human Performance, Icahn School of Medicine at Mount Sinai, New York, NY USA; 18 Department of Neurosurgery, Stanford Medical School, Stanford University, Stanford, CA USA; 19 Department of Physical Medicine and Rehabilitation, Santa Clara Valley Medical Center, San Jose, CA USA

**Keywords:** Health care, Medical research, Neurology, Neuroscience

## Abstract

Hypotension is an under-recognized consequence of spinal cord injury (SCI). Clinical assessments, such as head-up tilt testing, are resource-intensive and carry safety risks. In this study, we evaluated the validity of the hypotension symptom domain of the Autonomic Dysfunction Following Spinal Cord Injury (ADFSCI) questionnaire as a patient-reported outcome measure for hypotension-related symptoms in individuals with SCI. Known-groups validity and convergent validity were supported by higher hypotension symptom scores in participants with physiologically defined orthostatic hypotension (OH). Discriminative performance for OH classification was acceptable, and symptom frequency, severity, and contextual triggers were each associated with greater physiological BP instability. Symptom-level analyses identified blurred vision, light-headedness, fatigue, weakness, and dizziness correlated with blood pressure declines. To assess real-world applicability, we also analyzed a global dataset of patient-reported ADFSCI responses from individuals with SCI, enabling population-scale characterization of hypotensive symptom burden. In this dataset, higher symptom scores were associated with female sex and higher neurological injury level. Together, these findings provide converging evidence of supporting the validity of the hypotension symptom domain as a scalable measure of hypotension symptom burden in SCI for use in remote assessment and screening.

## Introduction

Hypotension is a common and debilitating condition across diverse clinical populations, including those with neurodegenerative diseases, diabetes, and spinal cord injury (SCI). Yet despite the impact of hypotension on brain perfusion, falls, and mortality, hypotension remains under-recognized and poorly monitored in clinical practice^1–9^. One subtype of hypotension, orthostatic hypotension (OH), is defined as a sustained drop in systolic (SBP) ( ≥ 20 mmHg) or diastolic (DBP) ( ≥ 10 mmHg) blood pressure (BP) upon transitioning to upright posture. Head-up tilt testing (HUTT) is the current gold standard for assessing OH, providing a controlled orthostatic challenge to evaluate autonomic cardiovascular regulation and BP instability ^5,10^. However, HUTT and similar laboratory-based assessments are resource-intensive, require specialized equipment, and carry risks such as tachyarrhythmia, presyncope, and syncope^11,12^. These limitations make them impractical for widespread screening, large scale trials, or real-world monitoring, particularly given the dynamic context-dependent nature of hypotension after SCI^13^. In daily life, symptoms of hypotension can occur during transfers, meals, or physical exertion, contexts that lab-based physiological testing cannot replicate^1–4,7^.

To address these gaps, patient-reported outcome measures (PROMs) have emerged as accessible tools for quantifying symptom burden directly from patients^14^. PROMs are now recognized by regulatory agencies such as the *Food and Drug Administration*, the *European Medicines Agency*, and *Health Canada* as critical for evaluating treatment benefit and patient experience^15^. However, few PROMs exist for monitoring hypotension-related symptom burden, and none are specifically validated for real-world assessments of hypotension symptoms in people with SCI. Existing instruments, such as the Orthostatic Hypotension Questionnaire (OHQ)^16^, were designed for ambulatory individuals and include items that may not be applicable or may be confounded in people with SCI. In contrast, the Autonomic Dysfunction Following Spinal Cord Injury (ADFSCI) questionnaire was specifically developed for individuals with SCI and incorporates clinically established triggers for hypotension^17^, including transfers, postprandial periods, and physical exertion, to capture the real-world frequency, severity, and functional impact of hypotension symptoms in daily life. However, the validity of the hypotension symptom domain of the ADFSCI has not yet been established.

Here, the aim of this study was to first evaluate the construct validity of the hypotension symptom domain of the ADFSCI questionnaire as a PROM that measures hypotension-related symptoms in people living with SCI, quantified as the hypotension symptom score (HSS). We evaluated internal consistency and known-groups validity between individuals with and without clinically defined OH using HUTT. Furthermore, we tested convergent validity against BP responses during HUTT. We quantified the discriminative performance of the hypotension symptom domain using receiver operating characteristic (ROC) analysis and logistic regression to evaluate utility for screening hypotension risk. Lastly, to evaluate broader applicability, we collected patient-reported data from individuals with SCI globally, enabling population-level analysis of hypotensive symptom burden. This global dataset also allowed us to identify sex-specific differences.

## Results

### Participants characteristics

**Fig. 1:**
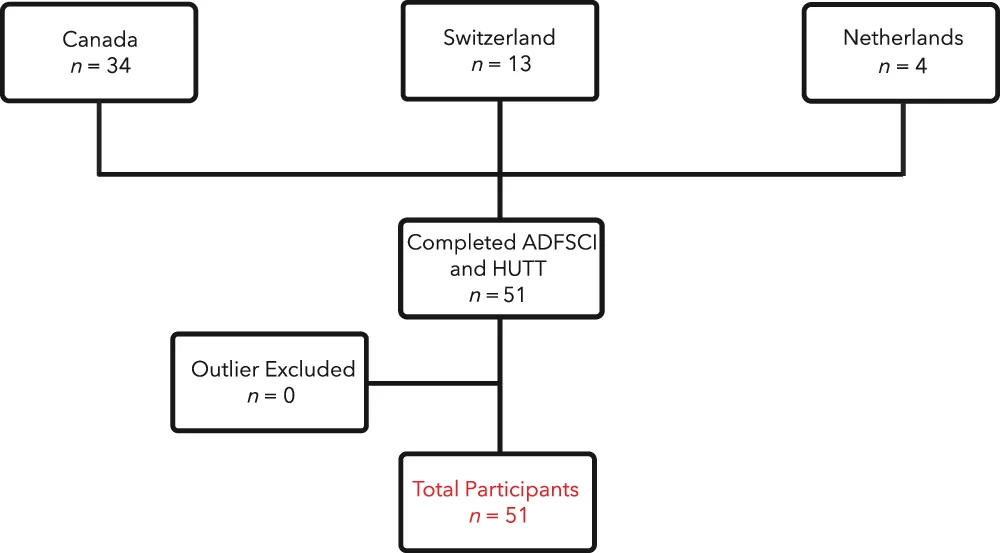
Participant flowchart. In-person assessment cohort characteristics. A total of 51 participants from three sites (Canada, *n* = 34, Switzerland, *n* = 13, The Netherlands, *n* = 4) completed both the ADFSCI questionnaire and HUTT.

A total of 51 individuals with SCI were recruited to evaluate construct validity of the ADFSCI hypotension symptom domain (the PROM) as a discriminative instrument for identifying hypotension-related symptoms (Fig. 1 and Table 1). The in-person assessment cohort had a mean age of 42.3 ± 14.4 years, included 29 males and 22 females, and consisted of cervical/thoracic injuries (44/7) with American Spinal Injury Association Impairment Scale (AIS) grades A–D (A: 25, B: 16, C: 7, D: 3). Clinically defined OH (OH + ) was present in 36 of 51 participants (70.6%).

**Table 1:** Participant characteristics

Characteristic	Value
Sample size (n)	51
OH+ (n/total)	36 / 51 (70.6%)
Age (years)	42.3 ± 14.4
Sex (male/female)	29/22
Injury level (cervical/thoracic)	44/7
AIS grade	A: 25, B: 16, C: 7, D: 3
Time since injury (years)	10.7 ± 12.6

Mean and standard deviation are reported in this table and all tables that follow.

*OH* orthostatic hypotension, *AIS* American Spinal Injury Association impairment scale.

### Internal structure: score distribution and internal consistency

The mean of HSS was 49.9 ± 36.7, ranging from 0 to 131, with 1 participant (2.0%) at the minimum score and 1 participant (2.0%) at the maximum score (Table 2), both well below the Consensus-Based Standards for the Selection of Health Measurement Instruments (COSMIN) threshold of 15% for relevant floor or ceiling effects. To assess internal consistency of the PROM, we calculated McDonald’s ω across 58 items. The PROM demonstrated high internal consistency (ω = 0.98, Table 2).

**Table 2:** Internal structure of hypotension symptom score

Property	Value
Number of items in the PROM	58
Mean HSS	49.9 ± 36.7
Range of HSS	0 – 131
Floor count (% at minimum score)	1 (2.0%)
Ceiling count (% at maximum score)	1 (2.0%)
McDonald’s ω (internal consistency)	0.98

*PROM* patient-reported outcome measure, *HSS* hypotension symptom score.

### Construct validity: known-groups and convergent validity

**Fig. 2:**
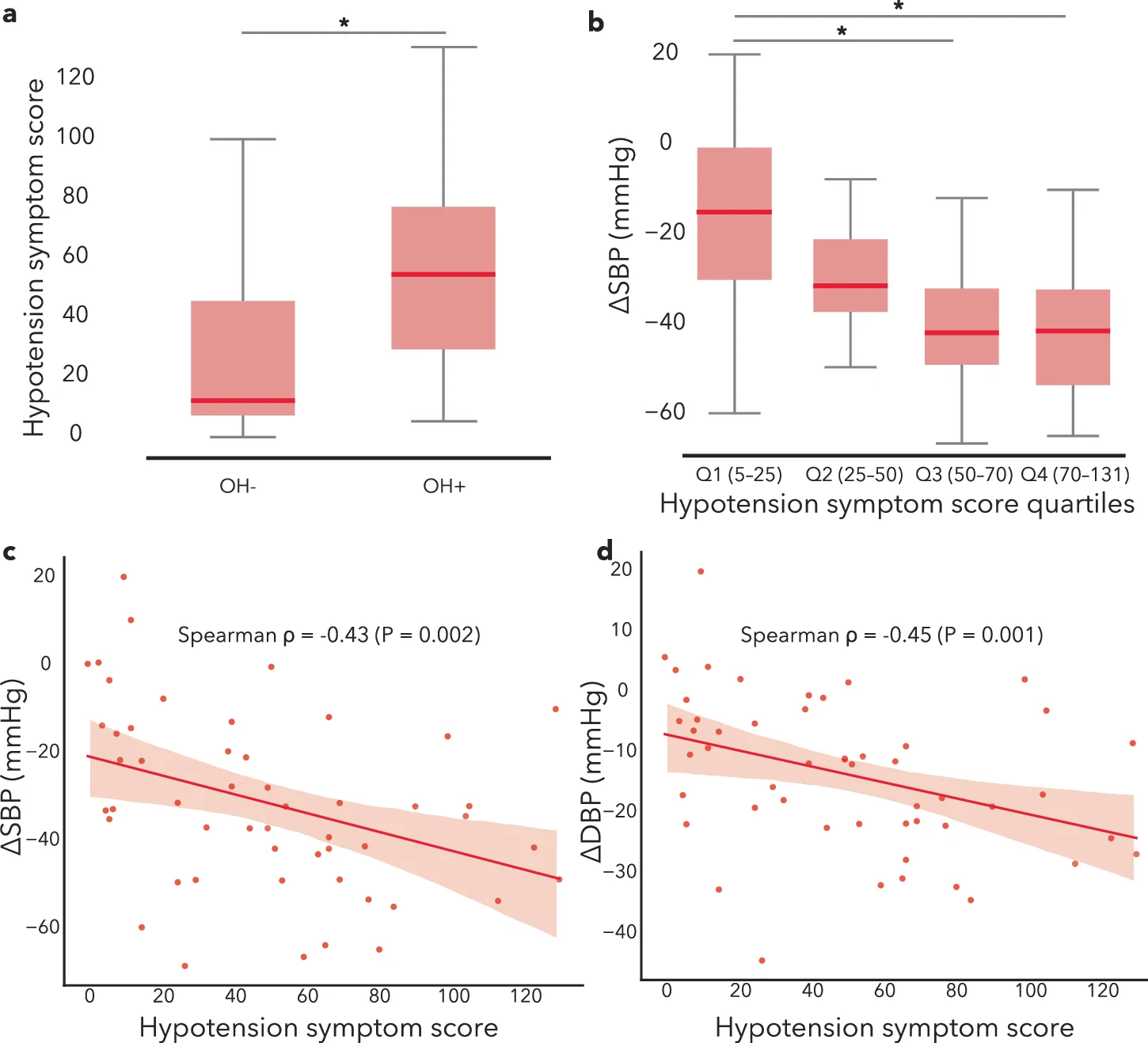
Hypotension symptom score correlates with blood pressure decline during HUTT. **a** Participants with OH+ show significantly higher HSS than OH- ones (Cohen’s d = 0.65, one-sided Mann–Whitney U test, *P* = 0.007). **b** Quartile analysis of HSS with ΔSBP. Significant difference among groups with ANOVA (*P* = 0.004). Q1 and Q3 are significantly different (Tukey post hoc *P* = 0.011). Q1 and Q4 are significantly different (Tukey post hoc *P* = 0.007). **c** Scatter plot with linear regression lines between HSS and ΔSBP (Spearman ρ = −0.43, *P* = 0.002). **d** Scatter plot with linear regression lines between HSS and ΔDBP (Spearman ρ = −0.45, *P* = 0.001). HSS significantly correlates with both ΔSBP and ΔDBP. Shaded areas represent 95% CIs for the regression lines. *Indicates *P* < 0.05.

Since the PROM and HUTT measure related, but not identical constructs, we hypothesized that OH+ group would report higher HSS than OH− group, with at least a moderate between-group difference (Cohen’s d ≥ 0.5). Consistent with the hypothesis, HSS was higher in the OH+ group than in the OH− group (56.8 ± 34.0 vs 33.5 ± 38.9), with a moderate between-group effect size (Cohen’s d = 0.65), and a statistically significant group difference (Mann–Whitney U test, *P* = 0.007, Fig. 2a).

To assess convergent validity with physiological orthostatic vulnerability, we specified a priori hypothesis that higher self-reported HSS would correspond to a larger orthostatic BP decline during HUTT. As HUTT and the PROM do not measure the same constructs, we further hypothesized that the strength of these associations would be moderate (expected Spearman ρ in the range between −0.30 and −0.50). Consistent with the hypothesis, when participants were stratified into quartiles by HSS, higher symptom-burden quartiles exhibited progressively larger SBP declines (ΔSBP), with a significant group difference (one-way ANOVA, *P* = 0.004, Fig. 2b). Tukey post hoc testing showed larger ΔSBP reductions in Q3 and Q4 compared with Q1 (Q1 vs Q3 *P* = 0.011, Q1 vs Q4 *P* = 0.007). In addition, HSS was moderately and inversely correlated with continuous HUTT BP changes, with higher HSS associated with larger drops in both SBP and DBP (Spearman ρ = −0.43, *P* = 0.002 for ΔSBP. ρ = −0.45, *P* = 0.001 for ΔDBP, Fig. 2c and d), indicating that greater symptom burden corresponded to more pronounced orthostatic BP decline during HUTT Table 3.

**Table 3:** Summary of validity tests

Property	Value
OH + / OH-	36 / 15
Mean HSS in OH+ group	56.8 ± 34.0
Mean HSS in OH- group	33.5 ± 38.9
Known-groups validity	Cohen’s d = 0.65 (95% CI: 0.04-1.27)
Mann–Whitney U test between groups	*P* = 0.007
Quartile analysis	ANOVA (*P* = 0.004) (Q1 v. Q3 *P* = 0.011 and Q1 v. Q4 *P* = 0.007)
Convergent validity (ΔSBP and HSS)	Spearman ρ = −0.43 (*p* = 0.002)
Convergent validity (ΔDBP and HSS)	Spearman ρ = −0.45 (*p* = 0.001)

*OH* orthostatic hypotension, *HSS* hypotension symptom score, *ANOVA* analysis of variance, *ΔSBP* change of systolic blood pressure, *ΔDBP* change of diastolic blood pressure.

### Discriminative performance for OH classification

**Fig. 3:**
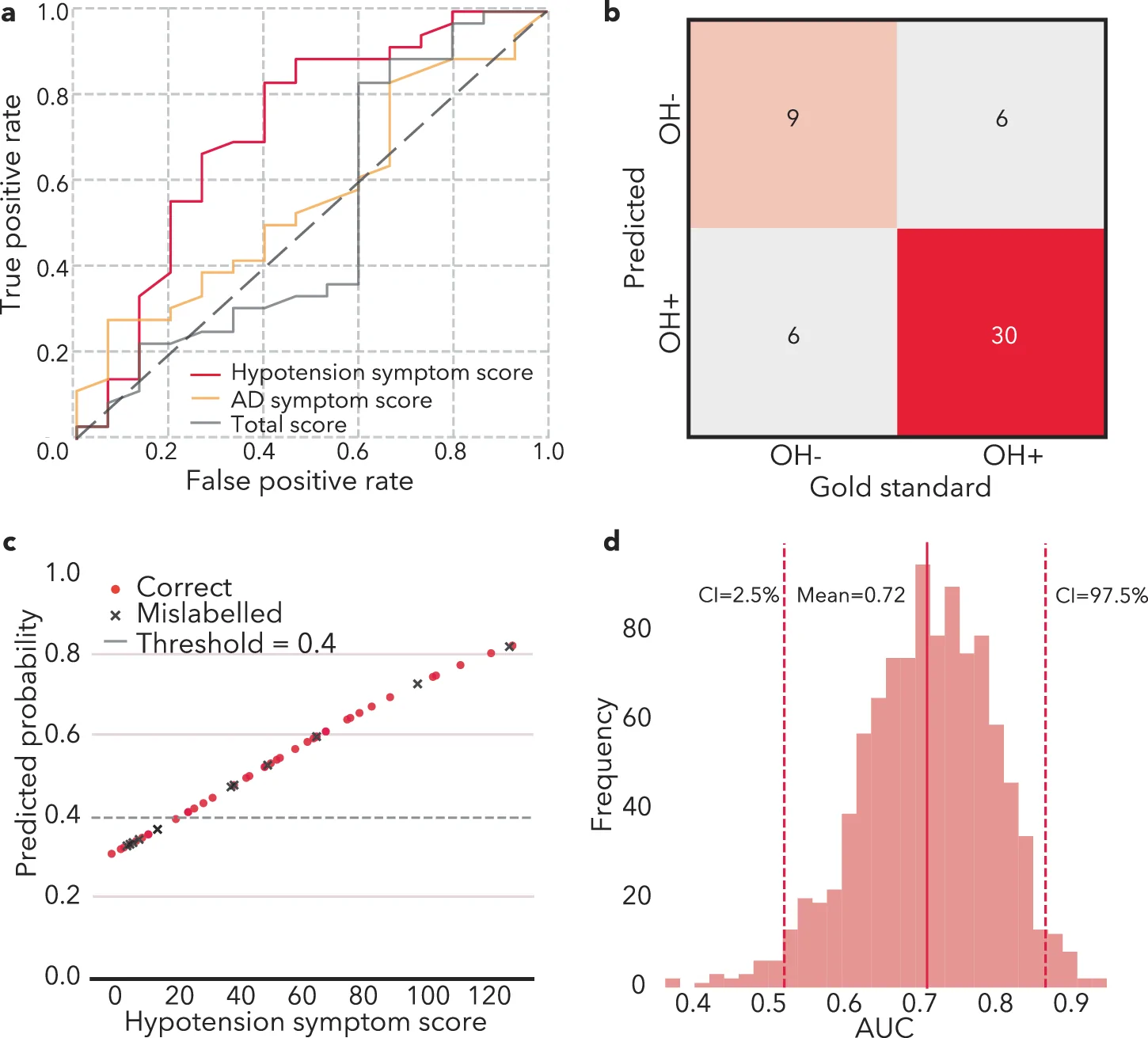
Discriminative ability of the PROM for clinically defined OH. **a** ROC demonstrates good performance using HSS (AUC = 0.72), better than the AD symptom score (AUC = 0.57) and the total score (AUC = 0.52). The model using the HSS demonstrates acceptable discrimination between individuals with and without OH. **b** Confusion matrix confirms classification accuracy of 0.76. Most participants with OH can be accurately identified using this model, minimizing missed cases. The specificity is 0.60 and the sensitivity is 0.83. **c** Classification plot shows correct and mislabeled participants using the HSS. **d** Bootstrapped AUC distribution confirms model robustness (95% CI: 0.53–0.88).

ROC analysis was used to evaluate how well the PROM distinguishes OH+ from OH- participants. Area under the curve (AUC) of the ROC using HSS was 0.72, indicating acceptable discriminative ability for classifying OH. This performance exceeded that of using the autonomic dysreflexia (AD) symptom score (AUC = 0.57) and the total score (AUC = 0.52) (Fig. 3a and Table 4). Further, the logistic regression achieved 0.76 accuracy, sensitivity of 0.83, and specificity of 0.60, F1 score of 0.83 (Fig. 3b and Table 4).

**Table 4:** Discriminative performance

Property	Value
Total participants	51
Gold standard OH+	36
Gold standard OH-	15
True positive *(OH* + *, predicted OH* + *)*	30
False negative (*OH* + *, predicted OH-)*	6
False positive *(OH-, predicted OH* + *)*	6
True negative *(OH-, predicted OH-)*	9
Accuracy *(TP* + *TN) / N*	0.76
Sensitivity *TP / (TP* + *FN)*	0.83
Specificity *TN / (TN* + *FP)*	0.60
False negative rate *FN / (TP* + *FN)*	0.17
False positive rate *FP / (TN* + *FP)*	0.40
F1 score *2TP / (2TP* + *FP* + *FN)*	0.83

*OH* orthostatic hypotension, *TP* true positive, *TN* true negative, *FP* false positive, *FN* false negative.

This performance is further evaluated in Fig. 3c, where the probability of OH is plotted against HSS, separating most correctly and incorrectly classified individuals. Misclassifications occurred predominantly among participants with low HSS. Model robustness was supported by bootstrapping (1000 samples), which yielded a mean AUC of 0.72 (95% CI: 0.53–0.88) (Fig. 3d).

### Construct validity of subcomponents (frequency, severity)

To examine whether the hypotension symptom domain subcomponents capture distinct aspects of symptom burden, items of the PROM were grouped into frequency (Q17, Q19–Q21, Q23) and severity (Q22 and Q24).

Since the PROM and HUTT do not measure the same construct, we hypothesized that individuals with clinically defined OH would report higher symptom frequency and severity scores than those without OH, with at least moderate between-group differences (Cohen’s d ≥ 0.5), and that both scores would be associated with larger ΔSBP declines during HUTT. Consistent with this hypothesis, symptom frequency scores were higher in OH+ than OH-, with a moderate effect size (Cohen’s d = 0.68), and a significant group difference (Mann–Whitney U test *P* = 0.007). Symptom frequency scores were also moderately inversely associated with ΔSBP (Spearman ρ = −0.43, *P* = 0.002, Supplementary Fig. 1a). Symptom severity scores showed a similar pattern, with higher scores in OH+ than OH- (*P* = 0.014) and a moderate effect size (Cohen’s d = 0.57). Symptom severity score also had a moderate inverse association with ΔSBP (Spearman ρ = −0.39, *P* = 0.005, Supplementary Fig. 1b).

### Exploratory analysis: contextual triggers and symptom-level associations

**Fig. 4:**
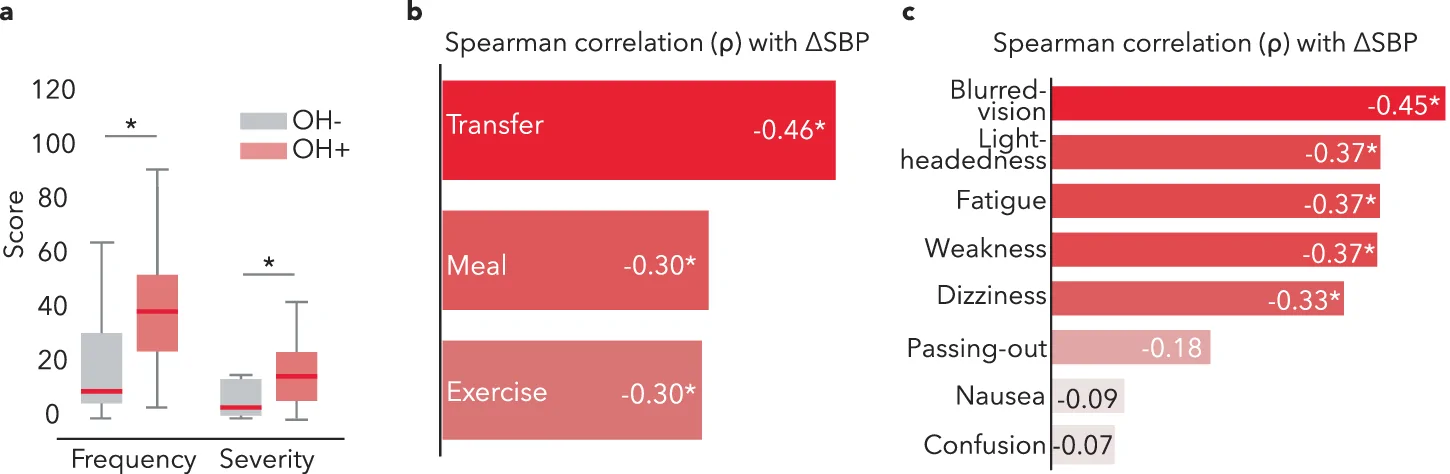
Sub-analysis of frequency, severity, and contextual triggers. **a** Scores of hypotension symptom frequency and severity were compared between participants with and without clinically defined OH. Boxplots show a significant difference between OH+ and OH- groups for frequency scores (*P* = 0.007), severity scores (*P* = 0.014). **b** Spearman correlation (ρ) between ΔSBP and individual contextual triggers. The asterisk indicates that the Spearman correlation is significant for transfers (ρ = -0.46, *P* = 0.001), meals (ρ = -0.30, *P* = 0.030), and exercise (ρ = -0.30, *P* = 0.034). **c** Spearman correlation (ρ) between ΔSBP and individual hypotension-related symptoms. Blurred vision (ρ = –0.45, *P* = 0.001), light-headedness (ρ = -0.37, *P* = 0.007), fatigue (ρ = -0.37, *P* = 0.007), weakness (ρ = -0.37, *P* = 0.007), and dizziness (ρ = –0.33, *P* = 0.017) were most strongly correlated with ΔSBP.

Contextual triggers (transfers, meals, and exercise) were assessed through activity-specific prompts (Q19–Q22) and correlated with ΔSBP. To examine the influence of specific contextual triggers, symptom scores corresponding to transfers, meals, and exercise were calculated by summing the scores of all items referencing the same trigger. These aggregated trigger-specific symptom scores were then used for Spearman correlation with ΔSBP. As shown in Fig. 4b, all three contexts, transfers (ρ = –0.46, *P* = 0.001), meals (ρ = –0.30, *P* = 0.030), and exercise (ρ = –0.30, *P* = 0.034), were moderately associated with ΔSBP. To identify symptom-level predictors of ΔSBP, Spearman correlation analyses were performed between each of the eight hypotension-related symptoms and ΔSBP (Fig. 4c). Blurred vision (ρ = –0.45, *P* = 0.001), light-headedness (ρ = –0.37, *P* = 0.007), fatigue (ρ = –0.37, *P* = 0.007), weakness (ρ = –0.37, *P* = 0.007), and dizziness (ρ = –0.33, *P* = 0.017) had moderate correlations with ΔSBP, whereas passing out, nausea, and confusion were not significantly correlated.

### Participant characteristics in a large global dataset

**Fig. 5:**
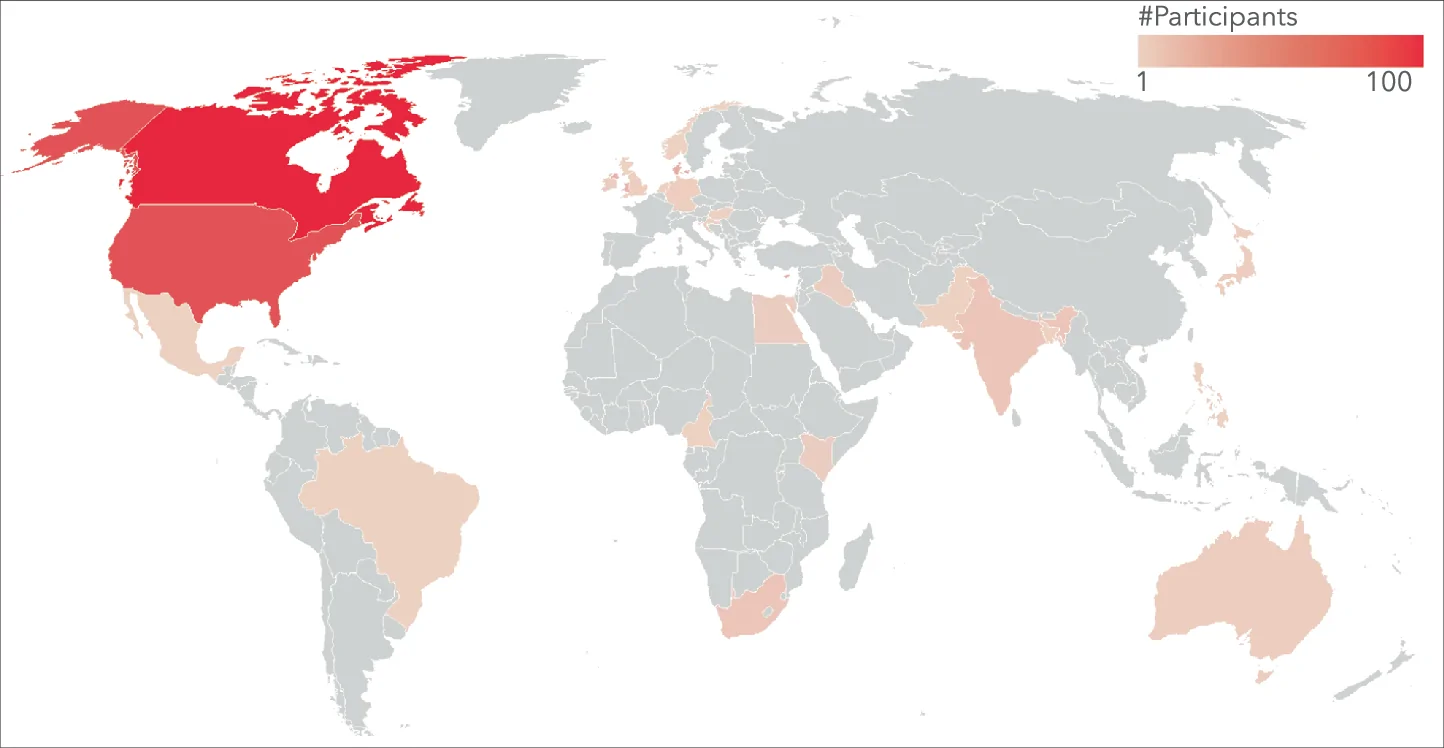
Distribution of the global dataset. Number of participants from 29 countries across the globe.

To explore whether the PROM captures plausible demographic and injury‑related patterns of hypotension symptom burden at scale, we collected a global dataset of 318 people (Fig. 5), comprising individuals from 29 countries. Applying 90% response completeness threshold yielded 159 valid responses for further analysis (Table 5). The mean HSS was 39.7 ± 33.4 (ranging from 4 to 162), indicating substantial inter-individual variability in patient-reported symptom burden. 51 participants (32.1%) self-reported experiencing OH. The mean age of participants was 44.3 ± 14.7 years, and the sample included 100 males and 59 females. Injury level distribution comprised 82 cervical, 58 thoracic, 15 lumbar, and 4 “other” injuries. With respect to neurological severity, 50 participants were classified as AIS A, 20 as AIS B, 12 as AIS C, and 10 as AIS D. AIS grade information was unavailable or classified as “other” for 67 participants. The mean time since injury was 18.6 ± 14.0 years.

**Table 5:** Participant characteristics of global dataset

Characteristic	Value
Total questionnaire collected (n)	318
Completeness threshold	90%
Valid sample size (n)	159
HSS	39.7 ± 33.4
Range of HSS	4.0–162.0
OH+ self-reported (n)	51
Age (years)	44.3 ± 14.7
Sex (male/female)	100/59
Injury level (C/T/L/Other)	82/58/15/4
AIS grade	A: 50, B: 20, C: 12, D: 10, Other: 67
Time since injury (years)	18.6 ± 14.0

*OH* orthostatic hypotension, *HSS* hypotension symptom score, *AIS* American Spinal Injury Association impairment scale.

### Construct validity of the global dataset: known-groups and convergent validity

To complement the in-person assessment cohort analyses and assess whether the same construct validity patterns were observed in a broader, geographically distributed SCI sample, we repeated the known-groups and convergent validity tests in the global dataset.

Since the PROM and HUTT do not measure the same construct, we hypothesized that individuals who self-reported OH (OH + ) would show higher HSS than individuals who did not report OH (OH − ), with at least a moderate between-group difference (Cohen’s d ≥ 0.5). Consistent with this hypothesis, OH+ participants (*n* = 51) showed higher HSS than OH- participants (*n* = 33) (Mann–Whitney U test, *P* < 0.001), with a large effect size (Cohen’s d = 1.41) (Supplementary Fig. 2a). Convergent validity was further supported by a moderate inverse association between HSS and resting SBP among participants with available data (*n* = 50, Spearman ρ = −0.43, *P* < 0.001, Supplementary Fig. 2b).

### Exploratory analysis: sex-specific symptom burden

In an exploratory multivariable logistic regression analysis, high symptom burden (top quartile of HSS) was associated with female sex (odds ratio (OR) 2.53, 95% confidence interval (CI) 1.23–5.24, *P* = 0.012), higher neurological level of injury (C1–T4 vs lower levels, OR 3.05, 95% CI 1.30–7.15, *P* = 0.010), self‑reported OH (OR 4.38, 95% CI 1.78–10.77, *P* = 0.001), and self-reported resting SBP < 100 mmHg (OR 2.59, 95% CI 1.16–5.80, *P* = 0.020) (Table 6).

**Table 6:** Logistic regression identifies variables associated with high hypotension symptoms

Variables	Hypotension symptom OR	95% CI	P
Sex (female)	2.53	1.23–5.24	0.012
Injury level (high)	3.05	1.30–7.15	0.010
Age ( ≥ 30)	1.14	0.45–2.90	0.781
Self-reported OH	4.38	1.78–10.77	0.001
Low self-reported resting SBP	2.59	1.16–5.80	0.020

*OR* odds ratio, *CI* confidence interval, *OH* orthostatic hypotension, *SBP* systolic blood pressure.

## Discussion

In this study, we evaluated the construct validity of the ADFSCI hypotension symptom domain as a discriminative PROM for quantifying hypotension symptom burden after SCI. We assessed known-groups and convergent validity by comparing HSS against hemodynamic responses measured during HUTT. The PROM demonstrated strong measurement performance, with minimal floor and ceiling effects (2.0%) and high internal consistency (ω = 0.98). Construct validity was supported by moderate between-group differences in participants with and without clinically defined OH, as well as moderate inverse associations between HSS and orthostatic BP decline. In addition, the PROM showed acceptable discriminative performance for OH classification (AUC = 0.72). We found that symptom frequency and severity contributed to OH discrimination, with higher scores associated with larger declines in SBP during HUTT. Moreover, known triggers of hypotension were moderately associated with larger SBP declines during HUTT, with transfers showing the strongest association. At the symptom level, blurred vision, light-headedness, fatigue, weakness, and dizziness demonstrated moderate associations with greater SBP decline. In our analysis of a global dataset, female sex and higher neurological level of injury were associated with greater symptom burden. Collectively, these findings provide converging evidence supporting the validity of the PROM for measuring hypotension symptom burden in SCI and support its feasibility for remote symptom assessment, while recognizing confirmatory physiological testing remains necessary.

SCI-related OH is predominantly neurogenic^5^, reflecting impaired descending sympathetic control and reduced peripheral vasoconstriction during upright posture, which causes a fall in arterial pressure. HUTT provides a controlled and reproducible orthostatic challenge that directly probes this impaired compensation^18^, making it an appropriate criterion measure for evaluating a symptom-focused PROM. In a previous study^19^, our group used a small cohort (*n* = 17) to compare symptom burden between clinically defined OH and non-OH groups, where OH status was determined by HUTT. Building upon this work, we aimed to obtain evidence evaluating the validity of the hypotension symptom domain of the ADFSCI questionnaire as a PROM for hypotension-related symptoms in people living with SCI and expanded the in-person cohort to 51 participants. To our knowledge, this is among the largest in-person cohort for clinical autonomic assessments after SCI^17,20^.

The in-person assessment cohort was enriched for individuals at higher risk of hypotension, with 70.6% (36/51) meeting clinically defined OH. This proportion closely mirrors the reported prevalence of OH in the SCI population (74%)^21^, providing an appropriate distribution of symptom burden for evaluating a hypotension-focused PROM. This in-person assessment cohort is also enriched in cervical injuries (44/51, 86.3%), and a high proportion of motor-complete injuries (AIS A/B: 41/51, 80.4%). Despite this enrichment, the cohort spanned a broad adult age range (42.3 ± 14.4 years) and included 29 males, 22 females (56.9%/43.1%). While this distribution enabled adequate representation of both sexes, the proportion of females (43.1%) exceeds that of the general SCI population (approximately 20% female)^22^, which may have contributed to the observed OH prevalence given known sex-related differences in cardiovascular autonomic function. This should be considered when interpreting generalizability.

The internal consistency of the PROM was high (McDonald’s ω = 0.98 across 58 items), which supports coherence but also potentially indicates some item redundancy. A previous study found that the HSS was not associated with OH detected from 24-hour ambulatory blood pressure monitoring (ABPM) recordings^17^. The lack of correlation may be explained by methodological challenges inherent to the 24-hour ABPM, including the inability to contextualize readings with respect to posture or activity, and intermittent sampling that may overlook transient or symptom-linked BP declines. In contrast, our study first evaluated known-groups validity of the HSS against the clinically defined with a moderate effect size (Cohen’s d = 0.65). We observed statistically significant inverse correlations between the HSS and both SBP and DBP declines (Spearman ρ = −0.43 for ΔSBP and ρ = −0.45 for ΔDBP). The magnitude between the PROM and clinically assessed OH after SCI was comparable to previous work validating the OHQ for assessing neurogenic OH in non-SCI populations^16^, supporting the construct validity of an SCI-specific instrument evaluated within its intended physiological context.

Our ROC analysis demonstrated that the PROM could distinguish individuals with and without clinically defined OH. The model achieved an AUC of 0.72, indicating acceptable discriminative accuracy for classifying physiologically assessed hypotension, and outperformed both the AD symptom score (AUC = 0.57) and the total questionnaire score (AUC = 0.52). This finding demonstrates the importance of domain-specific symptom constructs when developing PROM-based screening approaches. The model demonstrated an overall classification accuracy of 76%, correctly classifying 39 of 51 participants. Sensitivity was high (0.83), indicating that the majority of individuals with clinically defined OH were correctly identified by the PROM-based model. False negatives were relatively infrequent (*n* = 6, false negative rate = 0.17), whereas false positives occurred more commonly (*n* = 6, false positive rate = 0.40). The F1 score of 0.83 reflects a balance between precision and recall, suggesting that the model demonstrates moderate discriminative capacity under enriched conditions. Misclassifications predominantly occurred among participants with low hypotension symptom burden, which suggests that the model is most informative for identifying individuals with moderate to high symptom burden, rather than distinguishing subtle physiological differences near diagnostic thresholds. From a clinical screening perspective^23,24^, this error profile is appropriate for identifying hypotension risk in people with SCI, where minimizing missed cases is prioritized over excluding all false positives. Individuals who screen positive can be directed toward further physiological evaluation or monitoring, while the low false-negative rate reduces the likelihood that clinically relevant hypotension goes undetected. The in-person assessment cohort was enriched for higher-level and motor-complete injuries, therefore, performance may be overestimated relative to unselected SCI populations (including lower-level or motor-incomplete injuries). Overall, while not intended to replace clinical testing, the PROM offers a remote screening method to help identify individuals with higher risk of hypotension symptoms, particularly in settings where comprehensive physiological testing is not readily accessible.

Neurological symptoms are often heterogeneous and fluctuate over time^25^. PROM-based symptom burden assessment commonly separates frequency (how often symptoms occur) from severity (how intense they are) to better characterize symptom burden. In the in-person assessment cohort, both dimensions discriminated clinically defined OH status, with OH+ participants reporting higher hypotension symptom frequency (*P* = 0.007, Cohen’s d = 0.68) and severity (*P* = 0.014, Cohen’s d = 0.57) scores, suggesting that both components contribute to between-group differences and may be informative in addition to HSS. Symptom frequency and severity also demonstrated moderate convergent validity with ΔSBP during HUTT (Spearman ρ = −0.43 and ρ = −0.39, respectively), such that greater symptom burden corresponded to larger SBP declines. Contextual trigger analyses further supported the real-world relevance of this construct: trigger-specific symptom scores for transfers, meals, and exercise were each associated with larger orthostatic SBP declines (transfers: ρ = −0.46, *P* = 0.001, meals: ρ = −0.30, *P* = 0.030, exercise: ρ = −0.30, *P* = 0.034). Evidence from ambulatory BP monitoring studies in SCI similarly suggests that hypotensive episodes in daily life can cluster in predictable contexts, including the postprandial window and during routine activities such as transfers and physical activity, supporting the rationale for including contextual prompts within PROM items^6,25^. At the symptom level, blurred vision (ρ = −0.45, *P* = 0.001), light-headedness (ρ = −0.37, *P* = 0.007), fatigue (ρ = −0.37, *P* = 0.007), weakness (ρ = −0.37, *P* = 0.007), and dizziness (ρ = −0.33, *P* = 0.017) showed moderate associations with ΔSBP, suggesting these experiences may serve as particularly informative subjective markers of hypotensive vulnerability in SCI. Together, symptom-specific and trigger-specific items therefore strengthens the PROM’s ecological validity by connecting patient-reported burden to everyday contexts while maintaining objective linkage with standardized HUTT BP responses.

The global dataset extends the in-person findings by demonstrating that the PROM can be deployed at scale and showed clinically plausible demographic and injury-related patterns of symptom burden. Across 318 respondents from 29 countries, applying a 90% completeness criterion yielded 159 analyzable records, with wide variability in HSS (39.7 ± 33.4, range 4–162) and 32.1% self-reporting OH, supporting feasibility for remote capture of autonomic symptom burden across heterogeneous, international SCI populations. We further observed strong known-groups validity when stratifying participants by self-reported OH status. Individuals who reported OH (OH + , *n* = 51) had substantially higher HSS than those who did not report OH (OH − , *n* = 33) (Mann–Whitney U, *P* < 0.001), with a large between-group effect size (Cohen’s d = 1.41, 95% CI 0.92–1.89). Convergent validity was also supported by the association between symptom burden and available self-reported resting SBP data (*n* = 50), higher HSS was strongly associated with lower resting SBP (Spearman ρ = −0.43, *P* < 0.001), indicating that individuals reporting greater symptom burden also tended to exhibit a more hypotensive resting cardiovascular profile.

In the exploratory multivariable regression, higher neurological level of injury (C1–T4) was associated with greater odds of high symptom burden, consistent with known mechanisms of autonomic cardiovascular impairment after SCI^5^. Similarly, the associations with self-reported hypotension and low resting SBP ( < 100 mmHg) are directionally consistent with clinical observations that impaired sympathetic control, reduced vascular tone, and related contributors (e.g., low plasma volume, deconditioning) predispose individuals with SCI to hypotensive episodes, supporting the face validity of the PROM when deployed outside the laboratory^26,27^.

Female sex was independently associated with higher odds of greater hypotension symptom burden (OR 2.53, 95% CI 1.23–5.24), highlighting a potentially important sex-specific vulnerability. This finding extends previous reports that sex-related differences in cardiovascular risk exist after SCI by showing quantitatively that sex is associated with divergent symptom profiles^28–30^. These sex-related disparities merit mechanistic follow-up and prospective evaluation and highlight important future research directions. Sex differences in cardiovascular risk profiles and heart disease burden underscore the need to ensure that instruments assessing autonomic symptoms are sensitive to sex-specific experiences and potential differences in risk, triggers, and consequences. Future work should therefore interrogate mechanisms and factors underlying these differences (e.g., hormonal status, vascular regulation, medication patterns, and exposure contexts).

This work contains one of the largest in-person SCI cohorts combining a PROM assessment for hypotension symptoms with gold-standard orthostatic testing. However, we acknowledge that our sample was enriched for higher neurological levels of injury and more severe autonomic impairment. This potential bias is important to acknowledge, as our findings may not generalize to the entire SCI population. However, our findings do likely generalize to the cohort of individuals with SCI who would routinely be completing screening for hypotension after SCI, as hypotension is much more likely to be observed in those with higher-level and motor complete SCI^5,31,32^. Additionally, resting SBP values in the global dataset were self-reported, which may introduce measurement variability compared to standardized clinical assessment.

The ADFSCI questionnaire has been used to quantify symptoms of hypotension-related symptom burden after SCI^17,19^, however, as a PROM, it cannot directly measure hypotension itself. Instead, it captures perceived symptom burden that may reflect the impact of hypotension in daily life for people with SCI. As such, it may be appropriate to update the name of the ADFSCI to “Patient-reported outcome measure of cardiovascular dysfunction symptoms in people with spinal cord injury” (e.g., PROM-cSCI). Additionally, the data in this study was cross-sectional, which captures symptom burden and hypotension vulnerability at a single time point. Given that hypotension after SCI can fluctuate and evolve with recovery^26^, conditioning, and clinical management, longitudinal data will be critical to determine within-person responsiveness, test-retest reliability, and to evaluate treatment effects over time.

These limitations highlight the need for approaches that extend symptom assessment beyond in-person testing. The consistency of PROM performance across both clinically assessed and real-world populations supports scalable deployment. Although not used for data collection in the current study, a device-agnostic, cloud-based mobile health platform (Spine*Ally*) has been developed to enable remote collection of PROMs such as ADFSCI data alongside physiological signals in geographically distributed individuals living with SCI (Supplementary Fig. 3). Integration of PROM capture with wearable-derived physiological and activity signals is a particularly promising next step, because wearable technologies in SCI are increasingly recognized as a feasible approach for real-time behavioral monitoring and for tracking secondary complications and cardiovascular health in everyday settings. This multimodal infrastructure can strengthen ecological validity by linking symptoms to continuous cardiovascular/activity context, facilitate population-scale monitoring, and lay groundwork for multimodal risk stratification and more personalized management of hypotension after SCI^33,34^.

This study provides converging evidence supporting the validity of the ADFSCI hypotension symptom domain as a PROM of hypotension symptom burden after SCI. The domain demonstrated strong internal consistency, minimal floor and ceiling effects, and differentiated individuals with and without clinically defined OH. Exploratory analyses in a geographically diverse global cohort further demonstrated the broader applicability and scalability of the instrument and revealed plausible patterns of symptom burden, including a sex-specific difference. Collectively, these findings support the hypotension symptom domain of the ADFSCI as a scalable assessment tool for quantifying hypotension symptoms.

## Methods

### ADFSCI questionnaire

The ADFSCI questionnaire was developed by another group in 2014 using the Delphi technique to document participant-reported symptoms of autonomic dysfunction^17^. It contains four domains: demographics, medication usage, AD symptoms, and hypotension symptoms^17^. Demographics and medication items (Q1-6) served only as supporting participant characteristics and are not part of the PROM. The AD symptom domain (Q7-16) has been previously validated, and the summed score of these items is referred to as the AD symptom score. Items in the hypotension symptom domain (Q17-24) capture common symptoms (dizziness, light-headedness, blurred vision, nausea, weakness, confusion, fatigue, and the sensation of “passing out”) in relation to established contextual triggers such as transfers, meals, and exercise^5,17^.

In this study, we evaluated the hypotension symptom domain as the PROM that measures hypotension symptoms, defined as the frequency and severity of patient-reported symptoms associated with well-established cardiovascular stressors. The PROM is evaluated as a discriminative instrument, for use as a screening tool to quantify hypotension symptom burden, rather than as an evaluative instrument for longitudinal changes^35,36^. The HSS was calculated as the sum of scored items in this domain, with frequency assessed in Q17, 19, 20, 21, and 23, and severity is assessed in Q22 and 24. In addition to the total HSS, contextual-trigger–specific symptom scores for transfers, meals, and exercise were computed by summing all items that referenced the same trigger (Q19–22, details in Supplementary Table 1). The total ADFSCI score was computed as the sum of the HSS and the AD symptom score.

### Participants: In-person assessment cohort

All study procedures were conducted as part of four clinical safety and preliminary efficacy trials: STIMO-HEMO (ClinicalTrials.gov: NCT04994886; Lausanne University Hospital (CHUV)), HemON (ClinicalTrials.gov: NCT05111093; CHUV), HemON-NL (ClinicalTrials.gov: NCT05941819; Sint Maartenskliniek), and HEMO (ClinicalTrials.gov: NCT05044923; University of Calgary). The STIMO-HEMO study was approved by the Commission cantonale d’Ethique de la Recherche sur l’être humain (CER-VD 2021-00588; Swissmedic 10000882). The HemON study was approved by the Commission cantonale d’Ethique de la Recherche sur l’être humain (CER-VD 2021-D0025; Swissmedic 10000932). The HemON-NL study was approved by the Medisch-Ethische Toetsingscommissie Oost Nederlandse (METC 2023-16316) and the Centrale Commissie Mensgebonden Onderzoek (CCMO NL83694.000.23). The HEMO study was approved by the University of Calgary Conjoint Health Research Ethics Board (REB21-0027). Additional University of Calgary study procedures were approved by the University of Calgary Conjoint Health Research Ethics Board (REB21-0045). All trials and corresponding protocol amendments were conducted in accordance with the principles of the Declaration of Helsinki. Written informed consent was obtained from all participants prior to enrollment and participation in the studies.

51 participants who completed the questionnaire were deemed eligible for HUTT by meeting the following criteria: Age above 18 years with a diagnosis of a cervical or thoracic level SCI, AIS grade A–D, willing and able to provide informed consent, not pregnant, free of any pre-existing cardiac condition unrelated to SCI, and capable of attending in-person testing. Participants were recruited through clinician referrals, SCI community support organizations, and online web forms. Participants enrolled in the clinical trials were screened by research and clinical teams based on eligibility criteria.

### Protocol for HUTT and measurement of SBP and DBP

To assess orthostatic changes in BP, a HUTT was performed. Participants were instructed to abstain from caffeine, alcohol, cannabis, and blood pressure-modulating medications for 24 h prior to testing. To minimize the risk of AD, they were advised to avoid bowel routines within 5 h of testing and to empty their bladder immediately beforehand. The HUTT was conducted after completion of the ADFSCI questionnaire.

Participants were positioned supine on a motorized tilt table and secured with straps to ensure safety. Following a 5-to-10-minute silent baseline period with continuous BP recording, participants were tilted to 60° within 45 s. The tilt phase continued until the clinical definition of OH was met ( ≥ 20 mmHg decline in SBP or ≥10 mmHg decline in DBP) or a maximum of 10 minutes was reached, after which participants were returned to supine. These thresholds were pre-specified based on consensus definitions from both SCI and general clinical populations^37,38^.

Beat-by-beat arterial pressure was sampled at 200 Hz, while the SBP and DBP were extracted from the brachial-calibrated arterial pressure at 1 Hz (Finapres NOVA, Finapres Medical Systems B.V., Netherlands). Heart rate was sampled at 1 Hz. Supine BP was defined as the average of continuous BP recording over the 60 s preceding tilt. Tilt BP was calculated using a representative window around the minimum BP during tilt. The change in BP (ΔSBP and ΔDBP) was defined as the difference between the minimum tilted value and the supine value.

### Participants: global dataset

We created a global dataset to evaluate demographic and injury-related predictors of hypotension symptom burden in a heterogeneous SCI population. 318 individuals with a SCI aged 18 years or older who were fluent in English and able to provide informed consent completed the questionnaire. The dataset was developed as a digital survey-based cohort approved under REB21-0045 at the University of Calgary. Participants were recruited globally via targeted outreach through SCI community organizations across 29 countries. Resting SBP values in the global dataset were self-reported by participants.

### Outlier detection

Outlier detection was performed using the interquartile range (IQR) method. Participants were flagged as statistical outliers if their HSS exceeded 1.5 x IQR beyond the upper or lower quartiles. No outliers were detected. Missing questions were not addressed with any imputation during calculation. In the in-person assessment cohort, less than 5% of questions were missing across all included participants.

### Statistics

All statistical analyses were conducted using Python (v3.10) within Jupyter Notebook. No statistical methods were used to predetermine sample sizes, however, our sample sizes exceed those reported in previous publications^17,20^.

### Measurement properties

Following psychometric literature^36,39,40^, the hypotension symptom domain of the ADFSCI was evaluated as a discriminative instrument, for use as a screening tool to quantify hypotension symptom burden, rather than as an evaluative instrument of measuring longitudinal change. Accordingly, we focused on internal consistency, and construct validity (known-groups validity and convergent validity) and did not formally evaluate responsiveness, minimal clinically important difference, or minimal detectable change.

### Floor and ceiling effects

Floor and ceiling effects were examined to assess whether the hypotension symptom domain could discriminate across the range of symptom burden. The proportion of participants scoring at the minimum and maximum possible scores was then calculated. According to COSMIN criteria^39^, floor or ceiling effects were considered present if more than 15% of participants achieved the lowest or highest score, respectively. Absence of such clustering was interpreted as evidence that the hypotension symptom domain can distinguish between low, moderate, and high levels of hypotension symptom burden.

### Internal consistency

Internal consistency of the hypotension symptom domain was quantified using McDonald’s Omega (ω), which was calculated from a unidimensional factor model using the standardized factor loadings (λᵢ) and error variances (θᵢ) for each item:

$$\omega =\frac{{(\sum {\lambda }_{i})}^{2}}{{(\sum {\lambda }_{i})}^{2}+\sum {\theta }_{i}}$$

Values ≥ 0.70 were considered indicative of acceptable internal consistency, with ≥0.90 reflecting excellent homogeneity according to COSMIN criteria^39^.

### Known-groups validity

Known-groups validity was assessed as part of construct validity by testing the a priori hypotheses that participants with OH + , would report higher HSS than OH − , with at least a moderate between-group effect size (Cohen’s d ≥ 0.5). Group differences were evaluated using a Mann–Whitney U test, and effect size was quantified using Cohen’s d with 95% CIs. Confirmation of the hypothesized direction and magnitude of group differences was taken as evidence of known-groups validity^41^.

### Convergent validity

Convergent validity was evaluated as part of construct validity by testing the a priori hypothesis that higher HSS would be associated with larger orthostatic BP declines during HUTT. Specifically, we expected a moderate inverse association between HSS and maximal tilt-induced ΔSBP and ΔDBP (expected Spearman ρ from −0.30 to −0.50). Associations were examined using Spearman correlations (ρ) given the ordinal nature of HSS and potential non-normality of hemodynamic variables^40^.

### ROC analysis

To assess how well the hypotension symptom domain distinguishes participants *with* versus *without* clinically defined OH, we evaluated classification performance against an external clinical criterion (OH+ vs OH− based on HUTT). The probabilities of OH were obtained from a univariable logistic regression model with HSS as the sole variable. The AUC and its bootstrap distribution were estimated to summarize overall discriminative ability, with AUC values of 0.7–0.8 interpreted as acceptable and >0.8 as high. An optimal classification threshold was identified by maximizing accuracy, and corresponding sensitivity, specificity, and F1 score were reported.

### Odds ratio and logistic regression for the global dataset

As an exploratory analysis, multivariable logistic regression was applied to the global dataset to examine associations between participant characteristics and high symptom burden (defined as the top quartile of HSS). Participants missing more than 10% of responses within the hypotension symptom domain were excluded from the primary analysis. The following variables were a priori selected in the analysis based on clinical relevance^7,17,29,42–44^: sex (male vs female), neurological level of injury (high-level C1-T4 vs low-level T5 and below), self-reported OH (yes vs no), self-reported AD (yes vs no), and resting SBP categorized as low ( < 100 mmHg), normal (100-130 mmHg), or high ( > 130 mmHg), age ( ≥ 30 vs < 30 years)^44^. No variable was selected through data-driven procedures. ORs and 95% CIs were calculated for each variable and then adjusted where appropriate. ORs and 95% CIs are reported as measures of association. Statistical significance was defined as *P* < 0.05. One record with a physiologically implausible resting SBP value (SBP = 15 mmHg) was treated as an error in self-reporting and excluded from analyses involving resting BP in the global dataset.

## Supplementary information


Supplementary Information


## Data Availability

The datasets generated and analyzed during the current study are not publicly available due to privacy restrictions but are available from the corresponding author on reasonable request.

## Code availability

All statistical analyses were conducted using Python (v3.10) within Jupyter Notebook. The code used in this study is available at https://github.com/young24/Code-repo-for-ADFSCI-validation-paper.
