# Supplementary material for: Validating the ADFSCI hypotension symptom domain as a scalable patient reported outcome measure in spinal cord injury
Source: NPJ Digit Med. 2026 Jun 1;9:664. doi: 10.1038/s41746-026-02841-w (PMC13522484; doi:10.1038/s41746-026-02841-w)
Supplement: Supplementary file 1 [file 41746_2026_2841_MOESM1_ESM.pdf]

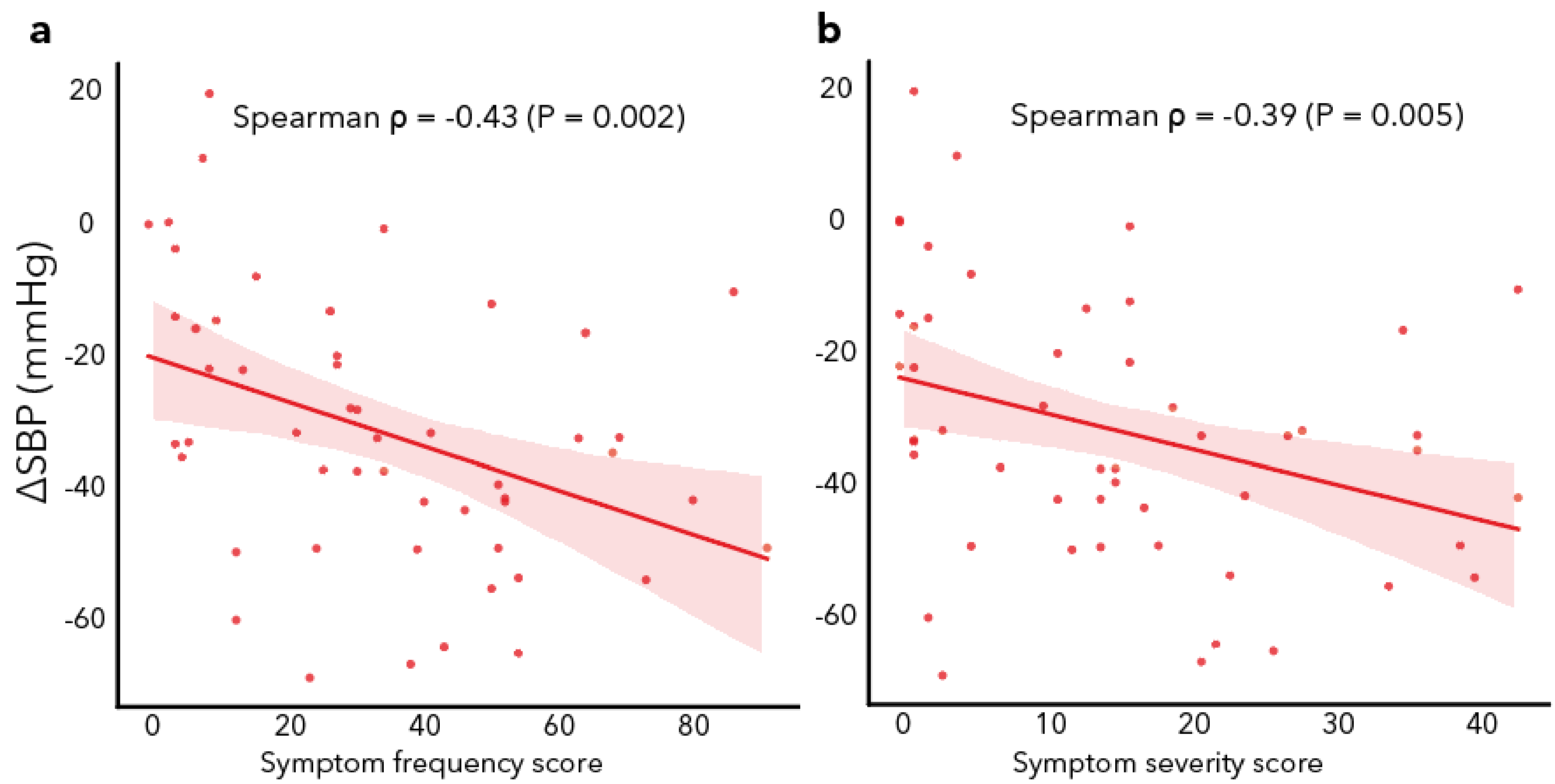

Supplementary Figure 1. Convergent validity of symptom frequency score and symptom severity score.
a) Spearman correlation between ΔSBP and symptom frequency score (ρ = −0.43, P = 0.002).
b) Spearman correlation between ΔSBP and symptom severity score (ρ = −0.39, P = 0.005).

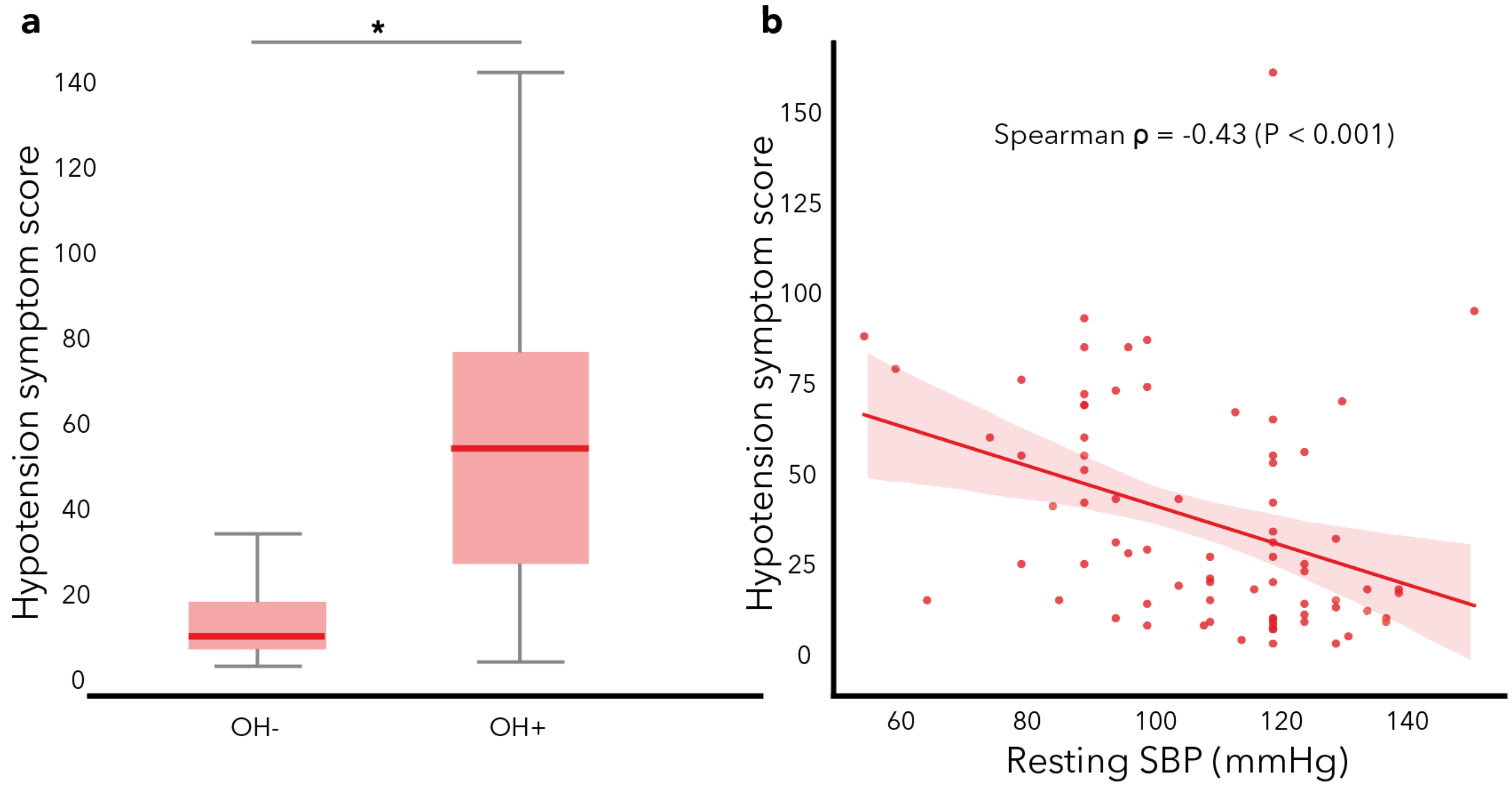

Supplementary Figure 2. Construct validity of the global dataset.
a) Known-groups validity analysis showing significantly higher HSS in individuals with OH+, compared with those with OH−, yielding a large between-group effect size (Cohen's d = 1.41, 95% CI 0.92–1.89).
b) Convergent validity analysis demonstrates a moderate inverse association between HSS and resting SBP (Spearman ρ = −0.43, n = 50).

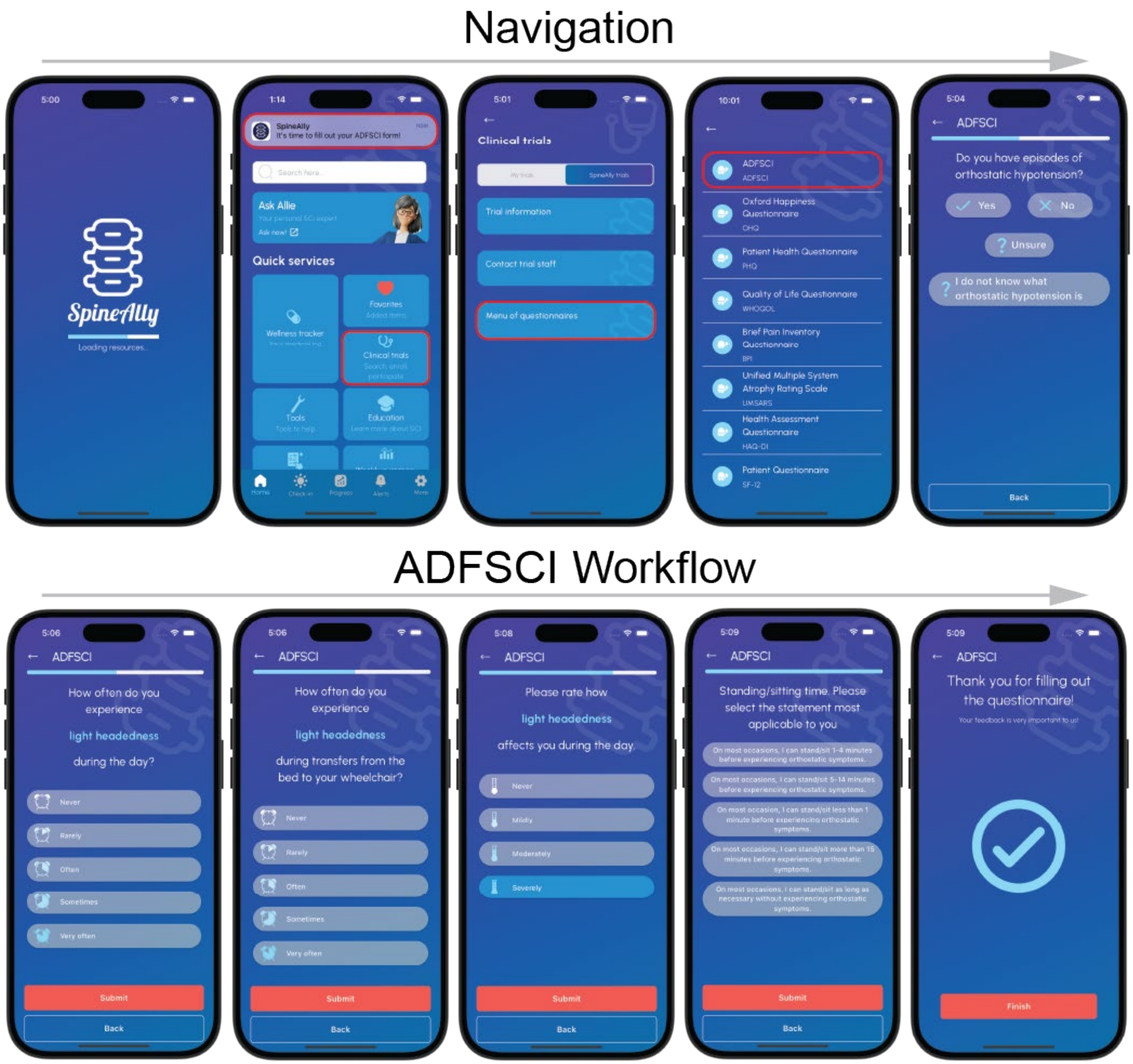

Supplementary Figure 3. SpineAlly and ADFSCI workflow.
Representative screenshots of Spine*Ally* for ADFSCI navigation user interface.

Supplementary Table 1. ADFSCI Scoring

Score/Item	Description	Points
**Hypotension - Severity**		
Q22_transfers	Please rate how much the following symptoms affect you **during transfers**.<br>• Dizziness<br>• Lightheadedness<br>• Blurred Vision<br>• Nausea<br>• Weakness<br>• Confusion<br>• Fatigue<br>• Passing Out	Never = 0<br>Mildly = 1<br>Moderately = 2<br>Severely = 3
Q22_meal	Please rate how much the following symptoms affect you **after a meal**<br>• Dizziness<br>• Lightheadedness<br>• Blurred Vision<br>• Nausea<br>• Weakness<br>• Confusion<br>• Fatigue<br>• Passing Out	Never = 0<br>Mildly = 1<br>Moderately = 2<br>Severely = 3
Q22_exercise	Please rate how much the following symptoms affect you **during or after exercise**<br>• Dizziness<br>• Lightheadedness<br>• Blurred Vision<br>• Nausea<br>• Weakness<br>• Confusion<br>• Fatigue<br>• Passing Out	Never = 0<br>Mildly = 1<br>Moderately = 2<br>Severely = 3

Q24	Standing/Sitting time. Please select the statement most applicable to you<br>• On most occasions, I can stand/sit as long as necessary without experiencing orthostatic symptoms<br>• On most occasions, I can stand/sit more than 15 minutes before experiencing orthostatic symptoms.<br>• On most occasions, I can stand/sit 5-14 minutes before experiencing orthostatic symptoms.<br>• On most occasions, I can stand/sit 1-4 minutes before experiencing orthostatic symptoms.<br>• On most occasion, I can stand/sit less than 1 minute before experiencing orthostatic symptoms.	As long as necessary without experiencing orthostatic symptoms = 1,<br>more than 15 minutes = 2,<br>5-14 minutes = 3,<br>1-4 minutes = 4,<br>less than 1 minute = 5.
**Hypotension - Frequency**		
Q17	How often do you experience the following symptoms during the day?<br>• Dizziness<br>• Lightheadedness<br>• Blurred Vision<br>• Nausea<br>• Weakness<br>• Confusion<br>• Fatigue<br>• Passing Out	Never = 0,<br>Rarely = 1,<br>Sometimes = 2,<br>Often = 3,<br>Very often = 4
Q19	How often do you experience the following symptoms during transfers from the bed to your wheelchair?<br>• Dizziness<br>• Lightheadedness<br>• Blurred Vision<br>• Nausea<br>• Weakness<br>• Confusion<br>• Fatigue<br>• Passing Out	Never = 0,<br>Rarely = 1,<br>Sometimes = 2,<br>Often = 3,<br>Very often = 4

Q20	How often do you experience the following symptoms after a meal?<br>• Dizziness<br>• Lightheadedness<br>• Blurred Vision<br>• Nausea<br>• Weakness<br>• Confusion<br>• Fatigue<br>• Passing Out	Never = 0,<br>Rarely = 1,<br>Sometimes = 2,<br>Often = 3,<br>Very often = 4
Q21	How often do you experience the following symptoms during or after exercise?<br>• Dizziness<br>• Lightheadedness<br>• Blurred Vision<br>• Nausea<br>• Weakness<br>• Confusion<br>• Fatigue<br>• Passing Out	Never = 0,<br>Rarely = 1,<br>Sometimes = 2,<br>Often = 3,<br>Very often = 4
Q23	Conditions under which orthostatic symptoms occur. Please select the statement most applicable to you.<br>• Never or rarely experience orthostatic symptoms under any circumstances<br>• I sometimes experience orthostatic symptoms under certain conditions, such as prolonged standing/sitting, a meal, exertion (e.g., walking/propelling a wheelchair), or when exposed to heat (e.g., hot day, hot bath, hot shower).<br>• I often experience orthostatic symptoms under certain conditions, such as prolonged standing/sitting, a meal, exertion (e.g., walking/propelling a wheelchair), or when exposed to heat (e.g., hot day, hot bath, hot shower).<br>• I usually experience orthostatic symptoms under certain conditions, such as prolonged standing/sitting, a meal, exertion (e.g., walking/propelling a wheelchair), or when exposed to heat (e.g., hot day, hot bath, hot shower).<br>• I always experience orthostatic symptoms when I stand up/sit up; the specific conditions do not matter.	Never = 1<br>Sometimes = 2,<br>Often= 3,<br>Usually = 4,<br>Always = 5
